# 796 Free Flaps in Acute Burns: A 12-Year Single-Center Experience and Systematic Review of the Literature

**DOI:** 10.1093/jbcr/irae036.337

**Published:** 2024-04-17

**Authors:** Francesco Egro, Jose Antonio Arellano, Hilary Liu, Mario Alessandri-Bonetti, Tiffany Jeong, Julia Kasmirski, Jenny A Ziembicki, Guy M Stofman

**Affiliations:** University of Pittsburgh Medical Center, Pittsburgh, PA; University of Pittsburgh Medical Center, Pittsburgh, PA; University of Pittsburgh Medical Center, Pittsburgh, PA; University of Pittsburgh Medical Center, Pittsburgh, PA; University of Pittsburgh Medical Center, Pittsburgh, PA; University of Pittsburgh Medical Center, Pittsburgh, PA; University of Pittsburgh Medical Center, Pittsburgh, PA; University of Pittsburgh Medical Center, Pittsburgh, PA

## Abstract

**Introduction:**

Free tissue transfer is usually considered a last resort in severe burn cases when local flaps are not a viable option. Since free flaps are seldom employed in burn reconstruction, there is a paucity of literature on the topic, especially in acute burn reconstruction. The aim of the study is to describe outcomes of burn patients requiring free tissue transfer at a single institution and review free flap failure in acute burn reconstruction reported in the literature, especially regarding how timing affects outcomes.

**Methods:**

A retrospective cohort study was conducted to review all patients who underwent free tissue transfer for burn-related injuries at a single institution between 2012 and 2023.

A systematic review and meta-analysis was conducted according to PRISMA guidelines and registered on the PROSPERO database (CRD42023404478). The following databases were accessed: Embase, PubMed, World of Science, and Cochrane Library. Studies reporting the free flap failure rate in burn patients were included.

**Results:**

12 patients required a free flap for their reconstruction. Ten flaps were performed during the acute care and two were performed for delayed reconstruction. The mean age was 44.8◻16.6 years, and the mean BMI was 25.8◻6.7. The mean follow-up time was 13.8◻14.6 months. The most common indication for free flap reconstruction was bone exposure (92%). The overall complication rate was 50%. Major complications included free flap loss (17%), hematoma (17%), infection (8%), venous thrombosis of the anastomosis (17%), and amputation (8%). 42% of the patients required re-operations.

In the literature, 454 free flaps were performed for acute burn reconstruction in 427 patients, with a total flap loss rate of 9.91% [95% CI: 7.48%, 13.02%]. Data on acute burn reconstruction timing from the injury was available for 275 flaps performed in 260 patients (88% male, 12% female). The pooled prevalence of free flap failure in three different time intervals ( < 5 days, 5-21 days, 22 days-6 weeks) was 7.32% [95% CI: 2.38% - 20.37%], 15.75% [95% CI: 10.70% - 22.59%], and 6.82% [95% CI: 3.10% - 14.36%], respectively.

**Conclusions:**

Free flap reconstruction carries a high risk of complications and should be considered only as a last resort for limb or life-threatening situations. However, the timing of the reconstruction appears to influence surgical outcomes. Free flap reconstruction performed between day and day 21 from burn injury had a trend towards higher flap loss rates.

**Applicability of Research to Practice:**

This research provides valuable insights into the applicability of free tissue transfer in burn reconstruction. This study demonstrates the high complication risk associated with free flap reconstruction and emphasizes the potential impact of timing on surgical outcomes, which can inform clinical practice and decision-making in severe burn cases.

